# Unpacking the benefits of black soldier fly frass fertilizer towards nematode suppression and potato production

**DOI:** 10.3389/fpls.2025.1509643

**Published:** 2025-02-10

**Authors:** Emmanuel O. Anedo, Dennis Beesigamukama, Benson Mochoge, Nicholas K. Korir, Solveig Haukeland, Xavier Cheseto, Moses Nyongesa, Patrick Pwaipwai, Sevgan Subramanian, Abdou Tenkouano, Betty Kibaara, Chrysantus M. Tanga

**Affiliations:** ^1^ International Centre of Insect Physiology and Ecology, Nairobi, Kenya; ^2^ Department of Agricultural Science and Technology, Kenyatta University, Nairobi, Kenya; ^3^ Tuber Crops Department, National Root Crops Research Institute, Umudike, Nigeria; ^4^ Division of Biotechnology and Plant Health, Norwegian Institute of Biotechnology (NIBIO), As, Norway; ^5^ Kenya Agricultural and Livestock Research Organization, Potato Research Centre Tigoni, Nairobi, Kenya; ^6^ Food Initiative, The Rockefeller Foundation, Africa Region Office, Nairobi, Kenya

**Keywords:** insect-derived chitin, potato yield, nematodes, regenerative agriculture, circular economy

## Abstract

Potato production is hindered by soil degradation and nematode infestation. Mineral fertilizers and synthetic nematicides are costly and cause negative impacts on humans and the environment, while organic fertilizers are less effective for soil health and nematode management. This study demonstrates the contribution of black soldier fly frass fertilizer (BSFFF) in nematode suppression and potato productivity when compared to commercial mineral fertilizer, organic fertilizer (SAFI), and nematicide. The on-farm experiments consisted of eight treatments: BSFFF, SAFI, BSFFF+5%chitin, NPK+nematicide, 50%BSFFF+50%NPK, 50%SAFI+50%NPK, 50%BSFFF+5% chitin+50%NPK, and control (unfertilized soil). Results revealed that all fertilizer treatments significantly increased potato growth, number of tubers (34 – 61%), and tuber yield (20 – 72%) relative to the control. Application of BSFFF+5% chitin produced 9 – 28% higher tubers per plant compared to other treatments. Over 26% higher tuber yield was achieved using BSFFF+5% chitin compared to NPK+nematicide treatment. Soil amendment with BSFFF+5% chitin caused 5–35% higher reduction in the number of cysts per 200 g soil^-1^ compared to NPK+nematicide and SAFI treatments. The same treatment reduced the PCN reproduction rate by 20% and 75% compared to NPK + nematicide and SAFI, respectively. Both BSFFF and NPK+nematicide treatments achieved comparable suppression of the number of eggs and infective juveniles (J2) per cyst^-1^ and eggs g^-1^ of soil. However, BSFFF+5% chitin reduced the number of eggs and J2 per cyst^-1^ and eggs g^-1^ of soil by 55–92% compared to SAFI. Our findings demonstrate that chitin-fortified BSFFF can significantly contribute to potato cyst nematode suppression and boost potato yields in smallholder farming systems, thus, making it a promising and sustainable alternative to commercial fertilizers and nematicides. Adopting this regenerative and multipurpose fertilizer will reduce reliance on synthetic fertilizers and nematicides, which are costly and harmful to the environment and human health.

## 1 Introduction

Globally, 33 – 52% of soils are affected by moderate and severe degradation, majorly due to soil erosion, salinization, acidification, and agricultural intensification (FAO and ITPS, 2015; ELD, 2015; Kopittke et al., 2019). The ongoing soil degradation is projected to reduce food production by 12% and increase food prices by 30% (ELD, 2015). In Africa for instance, approximately 83% of the arable land is degraded, with 75% severely depleted and lacking essential nutrients necessary for optimal crop growth and yield (Chianu et al., 2012; Wawire et al., 2021).

Currently, the productivity of African soils is declining due to land degradation, climate change, pest infestation, and poor soil and nutrient management practices, among others (Tully et al., 2015; Montanarella et al., 2016; Wortmann et al., 2019; ten Berge et al., 2019). To alleviate these challenges, judicious use of fertilizers and other regenerative agricultural practices to rejuvenate soil health and boost crop yields is necessary for the African farming system (Krah et al., 2019; Stewart et al., 2020; Gram et al., 2020). However, inorganic fertilizer use in sub-Saharan Africa is affected by high purchase costs, limited availability, reliance on imports, and weak fertilizer policies (Liverpool-Tasie et al., 2015; WFP, 2022). Additionally, the exclusive application of inorganic fertilizers induces nutrient imbalances, soil acidification, soil organic matter depletion, biodiversity loss, and dwindling yields of key food crops, including potatoes (Raimi et al., 2017).

Potato is an important food security crop in East Africa, providing income, nutrition, and sustenance to millions of people across the value chain (CIP, 2019; Otieno and Mageto, 2021). Interestingly, the average potato yield in many production areas has reduced drastically (Mugo et al., 2020; Price et al., 2021). In Kenya for instance, the average potato yield is 60 – 63% below the global average of 20 – 40 t ha^-1^ (VIB, 2019) and this can be attributed majorly to low soil fertility (Mugo et al., 2020), disease pressure, and soil-dwelling pests, especially potato cyst nematodes that affect over 80% of potato farms (Mwangi et al., 2015; Coyne et al., 2018a; Mburu et al., 2018, Mburu et al., 2020; Price et al., 2021). Potato cyst nematodes (Globodera rostochiensis and Globodera pallida) are recognized globally as invasive and quarantine pests of potato (Price et al., 2021). However, these pests are commonly overlooked due to their symptoms often mistaken for nutrient deficiencies, and the limited awareness of nematode challenges among smallholder potato farmers in sub-Saharan Africa (Coyne et al., 2018a). The multiplication rate of these nematodes has increased drastically across major potato farms mainly due to continuous potato cultivation on the same piece of land and the use of susceptible potato varieties (Mburu et al., 2020; Price et al., 2021). Historically, farmers have relied on synthetic nematicides for potato cyst nematode management, but these are costly and pose adverse health effects to humans and the environment. Therefore, there is a pressing need to explore sustainable and eco-friendly alternatives to address the biotic and abiotic challenges to soil health and potato productivity (Zhang et al., 2017; Sabarwal et al., 2018; Touray et al., 2021).

Research on the efficacy of chitin and chitin-rich organic amendments for the control of plant-parasitic nematodes (PPNs) attracted significant attention in the 1980s (Rodriguez-Kabana et al., 1987; Spiegel et al., 1988; Rodriguez-Kabana, 1986; Mian et al., 1982). This interest was driven primarily by their high nitrogen content, low carbon-to-nitrogen (C: N) ratio (Rodriguez-Kabana et al., 1987; Nico et al., 2004; Renčo et al., 2010; Oka, 2010) and stimulation of the activities of chitinolytic microorganisms which parasitize on nematode eggs and eggs sacks (Sarathchandra et al., 1996; Hallmann et al., 1999; Jin et al., 2005). These amendments also trigger a marked increase in soil chitinase activity which is closely linked to the enhanced activity of chitin-degrading microbes (Jung et al., 2002; Jin et al., 2005). Similarly, the production of volatile fatty acids and antibiotics (Akhtar and Malik, 2000; Ali et al., 2002; López-Robles et al., 2013), the release of nitrogenous compounds, organic acids, and other products of organic matter decomposition has been reported to contribute greatly to the effectiveness of chitin-rich organic soil amendments in plant-parasitic nematode control (Rodriguez-Kabana et al., 1987; Oka, 2010; Thoden et al., 2011).

Soil amendment with the chitin-rich black soldier fly frass fertilizer has been reported to increase the soil’s suppressiveness towards plant parasitic nematodes (Anedo et al., 2024; Kisaakye et al., 2024). The biocontrol efficacy of this fertilizer has been associated primarily with its high chitin content. For instance, the BSF pupal exuviae contains 10 – 30% chitin which is more cost-effective, highly soluble, and bioavailable (Hahn et al., 2020; Lagat et al., 2021). Besides the high chitin content, the low C: N ratio and high nitrogen content of the BSFFF helps in faster decomposition and subsequent release of ammonia and other by-products of organic matter decomposition associated with nematode control (Oka, 2010; Anedo et al., 2024). Also, BSFFF has been reported to induce plant systemic resistance against pests and pathogens and help in defense-related gene expression (Barragan-Fonseca et al., 2023; Li et al., 2023; Wantulla et al., 2023a). These findings are a game changer because insect chitin is more bioactive and cheaper than crustacean chitin which has been previously relied on for nematode management (Spiegel et al., 1988; Hussain et al., 2013);.

In the smallholder potato cropping systems of Africa and the tropics, the use of organic fertilizers is limited by long production time, low nutrient quality, chemical and biological contaminants, and competitive use of organic matter on the farm (Ndambi et al., 2019). Insect-driven recycling of organic wastes is emerging as an efficient, and eco friendly technology for the production of high quality organic fertilizers and biopesticides to complement mineral fertilizers and synthetic pesticides in soil health management and crop production (Beesigamukama et al., 2023a; Tanga and Kababu, 2023; Wantulla et al., 2023a, Wantulla et al., 2023b). This is because the organic fertilizer generated using insects also known as the insect frass fertilizer, contains plant nutrients that are readily available for plant uptake (Beesigamukama et al., 2020b, Beesigamukama et al., 2021; Menino et al., 2021), supplies plant growth hormones and boosts beneficial soil microbes (Fuhrmann et al., 2022; Barragan-Fonseca et al., 2022; Wantulla et al., 2023a). Soil amendment with black soldier fly frass fertilizer (BSFFF) has been found to increase the growth and yield of key food security crops such as maize (Beesigamukama et al., 2020a; Tanga et al., 2021), vegetables (Anyega et al., 2021; Abiya et al., 2022), bush beans (Chepkorir et al., 2024), and potato (Anedo et al., 2024). Application of insect frass fertilizer also induces systemic plant resistance against pests and pathogens, attracts pest parasitoids, and suppresses plant pathogens and pests (Barragan-Fonseca et al., 2022, Barragan-Fonseca et al., 2023; Wantulla et al., 2023a; Anedo et al., 2024)). Additionally, the pupae exuviae is an essential source of chitin which boosts the pesticidal properties of BSFFF and increases pathogen and pest suppression (Quilliam et al., 2020; Kemboi et al., 2022; Anedo et al., 2024).

The effectiveness of the chitin-fortified BSFFF in potato cyst nematode management has been demonstrated under greenhouse conditions, whereby soil amendment with BSFFF fortified with 5% chitin suppressed nematodes by up to 98% (Anedo et al., 2024). However, the performance of chitin-fortified BSFFF has not been validated under open field conditions, and its efficacy on nematode suppression and potato production in comparison to existing commercial organic fertilizers, mineral fertilizers, and nematicides has not been evaluated. This study, therefore, assessed comparatively, the nematicidal and fertilizer potentials of the BSFFF, commercial nematicide, inorganic fertilizer, and commercial organic fertilizer for potato production under open field conditions. The knowledge generated will support the use of chitin-fortified BSFFF as a cost-effective and regenerative fertilizer, and nematicide for enhancing the productivity of smallholder potato cropping systems.

## 2 Materials and methods

### 2.1 Study site

The study was carried out at the Kenya Agricultural and Livestock Research Organisation (KALRO) potato research center Tigoni station, central Kenya between December 2022 and August 2023. Tigoni station is located within the lower midland zone of Kiambu County (1°09’03.7”S and 36°41’08.3”E) at an elevation of 2131m above sea level. The region has a bi-modal rainfall pattern; the short rains begin in October and stop around December, while the long rains run from March to May. Annual rainfall in Tigoni is estimated to be about 1096 mm while the mean monthly temperature ranges between 12°C and 24°C with an average of 18°C. The soil is classified as Humic Nitisols, characterized by deep, well-drained, and reddish-brown friable clay with a pH ranging from 4.3 to 5.82 (Muthoni and Kabira, 2010; Rop et al., 2022). Before experiments, soil samples were collected randomly from the experimental site and bulked together to form a composite (Knowles and Dawson, 2018). A sub-sample was taken to the laboratory for determination of pH, nutrients (nitrogen, phosphorus, potassium, calcium, magnesium, sulphur, sodium, manganese, iron, copper, zinc, and boron), organic matter, exchangeable acidity, acid saturation, cation exchange capacity, and texture using standard methods (Okalebo et al., 2002). The results of soil analysis are presented in **Table 1**.

**Table 1:** Chemical properties of the experimental soil, black soldier fly frass fertilizer, chitin-fortified black soldier fly frass fertilizer, and SAFI organic fertilizer.

Parameter	Test soil	BSF frass fertilizer	Chititin-fortified BSF frass fertilizer	Commercial organic fertilizer (SAFI)
pH (H_2_O)	5.65	5.49	5.71	5.89
Electrical conduxtivity(mS/cm)	–	11.8	16.1	5.60
Organic carbon (%)	2.74	43.9	43.6	28.5
Nitrogen (%)	0.23	3.69	3.91	0.53
Available phosphorus (%)	0.01	1.54	1.43	0.11
Exchangeable potassium (%)	0.09	2.37	2.17	0.86
Exchangeable calcium (%)	0.11	1.00	2.17	0.23
Exchangeable magnessium (%)	0.02	0.59	0.61	0.12
Manganesse (ppm)	365	301	552	534
Iron (ppm)	92.1	5710	4780	3340
Zinc (ppm)	16.1	191	191	39.2
Copper (ppm)	2.77	31.8	42.1	4.84
Boron (ppm)	0.71	26.9	24.7	14.4
Exchangeable sodium (%)	0.01	0.31	0.28	0.02
Carbon/nitrogen ratio	11.9	11.9	11.2	53.8
Cation exchange capacity (Cmol kg^−1^)	16.5	–	–	–
Acid saturation (%)	1.77	–	–	–
Exchangeable acidity (meq/100g)	0.20	–	–	–
Sand (%)	18.7	–	–	–
Silt (%)	19.9	–	–	–
Clay (%)	61.4	–	–	–
Textural class	Clay	–	–	–

**Figure 1:**
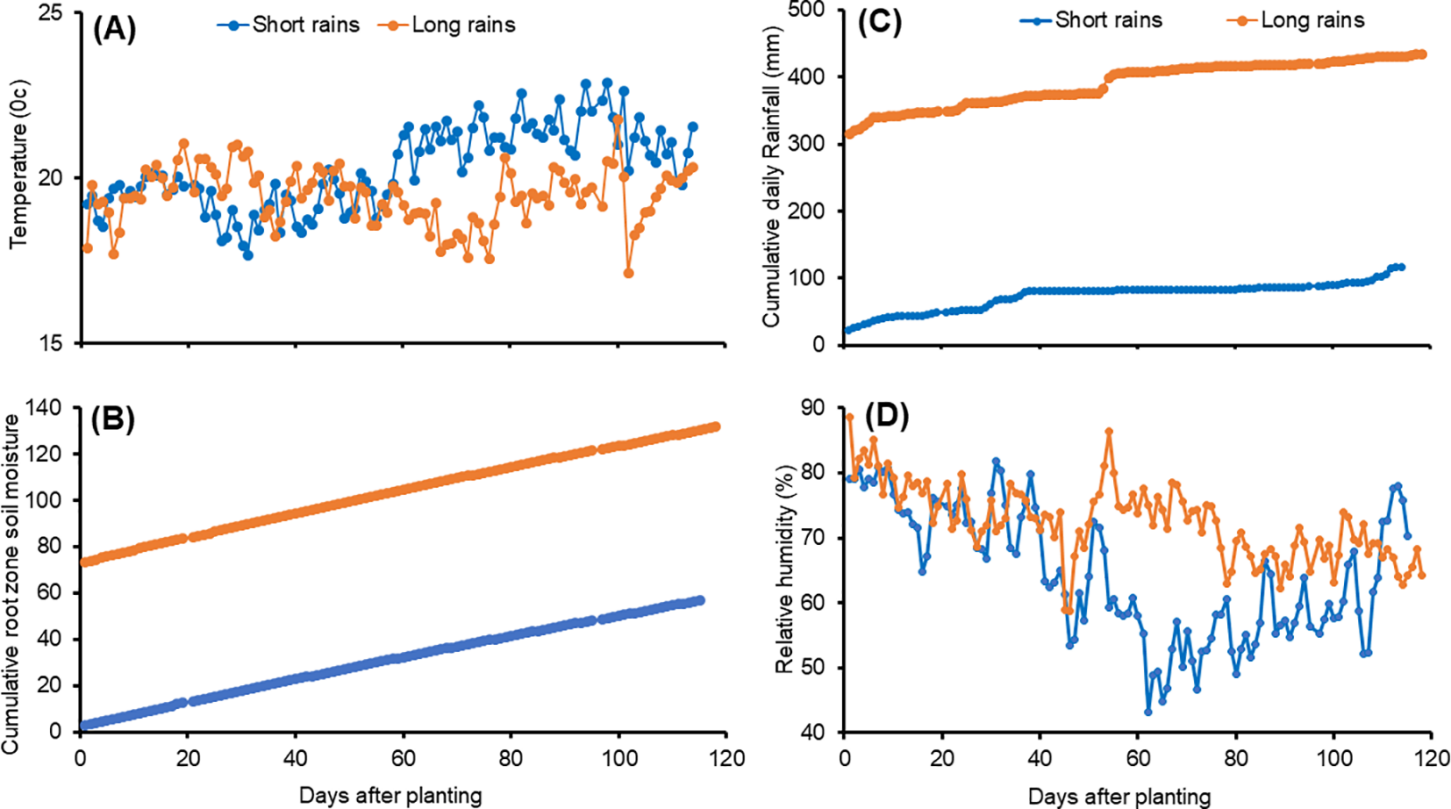
Daily temperature **(A)**, cumulative daily root zone soil moisture **(B)**, cumulative daily rainfall **(C)**, and relative humidity **(D)** of the study site during the experimental period.

During experiments, weather data was sourced from a weather station at Tigoni, while soil moisture data was obtained from the NASA Metrological database (https://power.larc.nasa.gov/data-access-viewer/). The average monthly temperatures were 17.9°C – 21.8°C during the short rains and 18.1 – 22.9°C during the long rains (**Figure 1A**). The cumulative root zone soil moisture was 2.8 –57.0 cm^3^ cm^-3^ and 73.3 – 131.7 cm^3^ cm^-3^ during the short and long rain seasons, respectively (**Figure 1B**). The cumulative daily rainfall was 23.2 –117.0 mm and 315.88 – 434.72 mm during the short rains and long rains, respectively (**Figure 1C**). The long rain season had higher relative humidity (64 – 75%) compared to the short rain season (53 – 75%) (**Figure 1D**). These values fall within the optimum temperature (15 – 20°C), relative humidity (50 – 85%), and annual rainfall (400 – 800 mm) recommended for potato production (Wheeler et al., 1989; Nyawade et al., 2019; National Potato Council of Kenya, 2020).

### 2.2 Sources of the experimental materials

The black soldier fly (BSF)-based chitin was obtained from BSF pupal exuviae sourced from a BSF colony maintained at the International Centre of Insect Physiology and Ecology (*icipe*), Nairobi, Kenya. The exuviae were washed using tap water, sun-dried for 4 days, and ground into a fine powder (< 2mm) using a KM-400 mechanical grinder (MRC laboratory equipment and manufacturing UK). The black soldier fly frass fertilizer (BSFFF) was obtained by feeding the black soldier fly larvae on the brewer’s spent grain (barley waste) according to the procedure outlined by Shumo et al. (2019). The larvae were harvested after two weeks, while the frass was composted for 5 weeks using the heap method described by Beesigamukama et al. (2020a). The commercial organic fertilizer (SAFI), a biochar-based organic fertilizer was sourced from Safi Organics Ltd., Mwea town, Kirinyaga County, Kenya. The biochar was produced using rice husk as feedstock at pyrolysis temperatures of 400°C and residence time of 3 hours. The mineral fertilizers, Muriate of potash (MOP), urea, and triple superphosphate (TSP) were sourced from Kenya Farmers’ Association (KFA) stores, Nairobi, Kenya. The BSFFF and SAFI were analyzed for nutrients and other chemical properties using standard laboratory methods described by Okalebo et al. (2002). The results are presented in **Table 1**.

### 2.3 Treatments and experimental setup

The experiment consisted of eight treatments: Black soldier fly frass fertilizer (BSFFF), applied at a rate equivalent to 150 kg N ha^-1^ (Otieno and Mageto, 2021), commercial organic fertilizer (SAFI), applied at a rate equivalent to 150 kg N ha^-1^, BSFFF in combination with 5% BSF chitin (BSFFF + 5chitin) as previously recommended in our sister studies (Anedo et al., 2024), NPK, BSFFF combined with NPK so that each supplies 50% (75 kg N/ha) of the total nitrogen (N) required (50BSFFF + 50NPK), SAFI combined with NPK so that each supplies 50% of the total N required (50SAFI + 50NPK), BSFFF+ 5% chitin combined with NPK so that each supplies 50% of the total N required (50BSFFF+ 5chitin + 50NPK), and control (unamended soil). The quantities of BSFFF, BSFFF + 5% chitin, and SAFI required to supply the nutrients were 4.07 t ha^-1^, 3.84 t ha^-1^, and 28.3 t ha^-1^, respectively. The NPK fertilizer was applied at the rate of 150 kg N ha^-1^, 60 kg P ha^-1,^ and 250 kg K ha^-1^ (urea 326.1 kg ha^-1^, triple super phosphate 298.7 kg ha^-1^ and muriate of potash fertilizers (1004.3 kg ha^-1^), respectively. The nematicide was applied at a company-recommended rate of 0.625 l ha^-1^. The organic and mineral fertilizers were applied at planting time using the band placement method, effected by placing the fertilizers in 5 cm deep bands that were covered with soil before planting. The commercial nematicide (Velum Prime from Bayer crop sciences, UK) was applied during planting. Pre-sprouted seeds of potato variety cv. shangi were planted on a 4m × 4m plot with a plant spacing of 75 × 30cm. The experiment was laid out in a randomized complete block design with four replications, and conducted for two cropping seasons. The treatments were applied every season. The experiments were managed using standard agronomic practices recommended for potato production. Ridomil gold (Syngenta East Africa Ltd.) was applied at 3 and 10 weeks after planting to control early and late blights.

### 2.4 Potato growth and yield

Data on leaf growth, number of branches, plant height, stem diameter, and leaf chlorophyll concentration were collected biweekly using 10 randomly selected plants from each plot, from the 4^th^ to the 12^th^ week after planting. Plant height was determined by using a tape measure, starting from ground level to the apex of the plant. The number of leaves was determined by counting fully developed and photosynthetically active leaves. The number of branches was determined by counting the number of fully developed branches per plant. Leaf chlorophyll concentration was determined using a chlorophyll meter (SPAD-502plus, Konica Minolita, Japan) placed on six fully developed leaves from the top. Stem diameter was measured using a vernier caliper (150 mm, Toolstream Ltd., UK) placed 10 cm above the soil surface.

Harvesting was conducted at 115 days after planting when the plants had reached physiological maturity indicated by the yellowing of the leaves and withering of stems. The plants from each plot were carefully uprooted to retrieve the tubers using a garden fork. The harvested tubers were separated from the shoots manually, counted, and graded into marketable and non-marketable tubers. The number of marketable tubers (i.e., those that were pest and disease-free and weighing above 25g) per plot was determined by hand counting. The marketable tuber weights were measured using an electronic weighing balance and used to determine the yields in tonnes per hectare [t ha^-1^]) (Asnake et al., 2023).

### 2.5 Soil nematode dynamics

Before planting and after harvesting, composite soil samples were collected from each plot using a systemic sampling pattern, mixed thoroughly, and a sub-sample of 200g was taken for cyst extraction (Coyne et al., 2018b). The soil samples were air-dried for 7 days, and the cysts were extracted using the Fenwick can flotation method (European and Mediterranean Plant Protection Organisation, 2013). The extracted cysts were collected on milk filter paper, air-dried, and manually picked using entomological forceps under a LEICA EZ4 stereomicroscope (Leica Microsystems GmbH. Germany). The viability of the cysts was assessed using Nile blue stain (Sigma Aldrich, USA), and 20 uniform-sized cysts (in triplicates) were selected for assessment (Mburu et al., 2020). After incubating the cyst with 0.01% Nile blue stain for 48 hours, the cysts were crushed to expose the viable eggs, non-viable eggs, and second-stage juveniles (J2). The stained eggs (non-viable), non-stained eggs (viable), and live (viable) second-stage juveniles (J2) were identified using a LEICA M80 stereomicroscope at ×40 magnification (Leica Microsystems GmbH. Germany). The cyst fertility (CF) was determined by adding the number of J2s, stained eggs, and non-stained eggs. Cyst viability (CV%) was determined using Equation 1 (Mburu et al., 2020).


(1) $$\text{Cyst viability}(\%)=\frac{\text{number of J}2\text{s}+\text{number of non}-\text{stained eggs}}{\text{Cyst fertility }}\times 100$$

The effectiveness of the fertilizer treatments in PCN suppression was determined by calculating cyst viability (Equation 1) and nematode multiplication rate (Equation 2).


(2) $$\text{Rf}=\frac{Pf}{\text{P}i}$$

Where:

*Pf* is the nematode population after harvesting and *Pi* is the nematode population before planting (Vanitha et al., 2019).

### 2.6 Statistical analysis

The normality of the collected data was assessed using the Shapiro-Wilk test. Subsequently, a linear mixed-effect model, utilizing the ‘lmer’ function from the ‘lme4’ package, was employed to analyze the interactive effect of the fertilizer treatments on potato growth parameters (plant height, number of leaves, number of branches, stem diameter, and leaf chlorophyll). This model treated fertilizer treatments and sampling time as fixed effects, while replication was considered a random effect. Furthermore, one-way analysis of variance was used to analyze the growth parameters for each sampling time, number of marketable tubers, tuber yield, and nematode population parameters. Least squares means were computed with the “lsmeans” package, and significant differences were determined using the “Tukey” test at a significance level of p ≤ 0.05. To explore the relationship between potato growth, yield, and nematode population densities, principal component analysis (PCA) was executed using the “prcomp” function from the “ggbiplot” package, pooling data from both seasons. All statistical analyses were performed using R software (R Core Team, 2024).

## 3 Results

### 3.1 Effect of chitin-fortified BSFFF and commercial fertilizers and nematicides on potato growth

#### 3.1.1 Plant height

**Figure 2:**
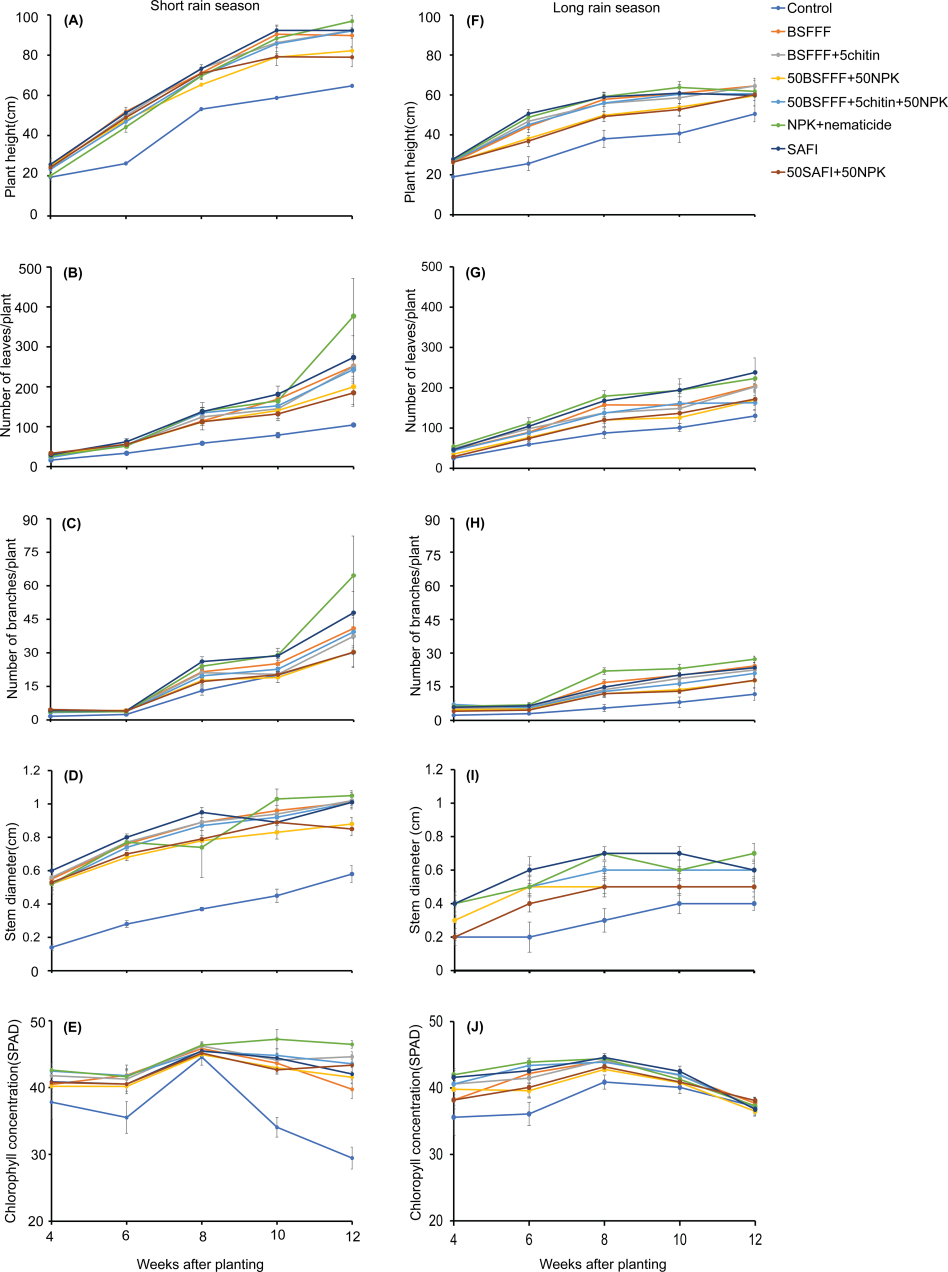
Effect of BSF frass fertilizer, commercial organic fertilizer, and NPK treatments on potato growth: plant height **(A , F)**, number of leaves, **(F, B, G)** number of branches **(C, H)** stem diameter **(D, I)** and chlorophyll concentrations **(E, J)** during the short rain season **(A–E)** and long rain season **(F–J)** experiments. Control, no fertilizer amendment; BSFFF, black soldier fly frass fertilizer applied at a rate equivalent to 150 kg N ha^-1^; BSFFF+5chitin, black solder fly frass fertilizer applied at a rate of 150kg N ha ^1^ and fortified with 5% chitin from BSF exuviae; 50BSFFF+50NPK, 50%black soldier fly frass fertilizer+50%NPK; 50BSFFF+5chitin +50NPK, 50%BSFFF+5%chitin+50%NPK; SAFI, Commercial organic fertilizer applied at a rate equivalent to 150 kg N ha^-1^; 50SAFI+50NPK, 50%SAFI+50%NPK; NPK+nematicide, NPK+ commercial nematicide. Per panel, means (± standard error) followed by the same letters are not significantly different at p < 0.05.

The potato height was significantly *(P* < 0.001) influenced by the different fertilizer treatments (short rain season: χ2 = 101.5, df =7, *P* < 0.001, long rain season: χ2 = 152.0, df =7, *P* < 0.001) and potato growth stage (short rain season: χ2 = 1452.0, df =4, *P* < 0.001, long rain season: χ2 = 794.3, df =4, *P* < 0.001) (**Figures 2A , F**). The interaction of the fertilizer treatments and potato growth stage was not significant during both seasons (short rain season: χ2 = 37.9, df =28, *P*=0.10, long rain season: χ2 = 28.8, df =28, *P* = 0.42). Compared to the control, soil amendment with the fertilizer treatments significantly (*P* < 0.001) increased the plant height by 22 – 32% and 23 – 34% during the short and long rains, respectively. However, there were no significant differences between BSFFF-based fertilizer sources, SAFI, and NPK + nematicides during both seasons.

#### 3.1.2 Number of leaves and branches

There were significant differences in the number of leaves of potatoes due to fertilizer treatments (short rain season: χ2 = 51.3, df =7, *P* < 0.001, long rain season: χ2 = 103.1, df =7, *P* < 0.001) and potato growth stage (short rain season: χ2 = 424.9, df =4, *P* < 0.001, long rain season: χ2 = 539.8, df =4, *P* < 0.001). The interaction effect of the fertilizer treatments and potato growth stage was significant during the short rain season only (short rain season: χ2 = 56.4, df =28, *P* < 0.01, long rain season: χ2 = 23.6, df =28*, P*=0.70) (**Figures 2B, G**). The fertilizer treatments increased number of leaves by 43 – 61% and 24 – 47% during the short and long rain seasons, respectively, compared to the control.

The potato branches were significantly increased by the different fertilizer treatments (short rain season, χ2 = 31.9, df = 7, *P* < 0.001, long rain season, χ2 = 158.2, df = 7, *P* < 0.001), potato growth stage (short rain season: χ2 = 456.4, df = 4, *P* < 0.001, long rain season: χ2 = 539.8, df = 4, *P* < 0.001) and their interaction (short rain season: χ2 = 41.2, df = 28, *P* < 0.1, long rain season: χ2 = 50.0, df = 4 = 28, *P* < 0.01) (**Figures 2C, H**). The fertilizer treatments increased the potato branches by 11– 46% and 41 – 69% during the short and long rains, respectively, compared to the control. In both seasons, soil amendment with NPK + nematicides achieved the highest increase in the number of branches which was 23 – 40% significantly higher than the values achieved using other treatments.

#### 3.1.3 Stem diameter and leaf chlorophyll content

The potato stem diameter increased significantly due to the fertilizer treatments (short rain season: χ2 = 413.3, df = 7, *P* < 0.001, long rain season: χ2 = 140.7, df = 7, *P* < 0.001) and potato growth stages (short rain season: χ2 = 422.1, df = 4, P < 0.001, long rain season: χ2 = 141.6, df = 4, *P* < 0.001). There was no significant difference between the fertilizer treatments and the potato growth stages during the short rain season (χ2 = 23.0, df = 28, *P* = 0.73) and long rain season (χ2 = 10.7, df = 28, *P* = 1.0), respectively (**Figures 2D, I**). Soil amendment with the fertilizer treatments significantly increased (*P* < 0.001) the potato stem diameter by 51 – 59% and 24 – 46% compared to the control during the short and long rains respectively. In both seasons, soil amendment with SAFI achieved the highest stem diameter, which was significantly higher than those of 50BSFFF + 50NPK and 50SAFI + 50NPK.

The potato leaf chlorophyll concentration varied significantly due to the fertilizer treatments (short rain season: χ2 = 194.4, df = 7, *P* < 0.001, long rain season: χ2 = 73.7, df = 7, *P* < 0.001), potato growth stage (short rain season: χ2 = 121.3, df = 4, *P* < 0.001, long rain season: χ2 = 234.1, df = 4, *P* < 0.001) and their interactions (short rain season: χ2 = 91.3, df = 28, *P* < 0.001, long rain season: χ2 = 50.5, df = 28, *P* = 0.01) (**Figures 2E, J**). An increase in the potato leaf chlorophyll concentration by 13 –19% and 5 – 7% compared to the control during the short and long rains, respectively was observed. In both growing seasons, NPK + nematicides achieved the highest increase in the potato leaf chlorophyll concentration which was significantly different from other fertilizer sources by 5 –7% during the short rains. The leave chlorophyll concentration started dropping from the 8^th^ week down to the 12^th^ week after planting.

### 3.2 Yield of potatoes grown in soil amended with chitin-fortified BSFFF and commercial fertilizers and nematicides

**Figure 3:**
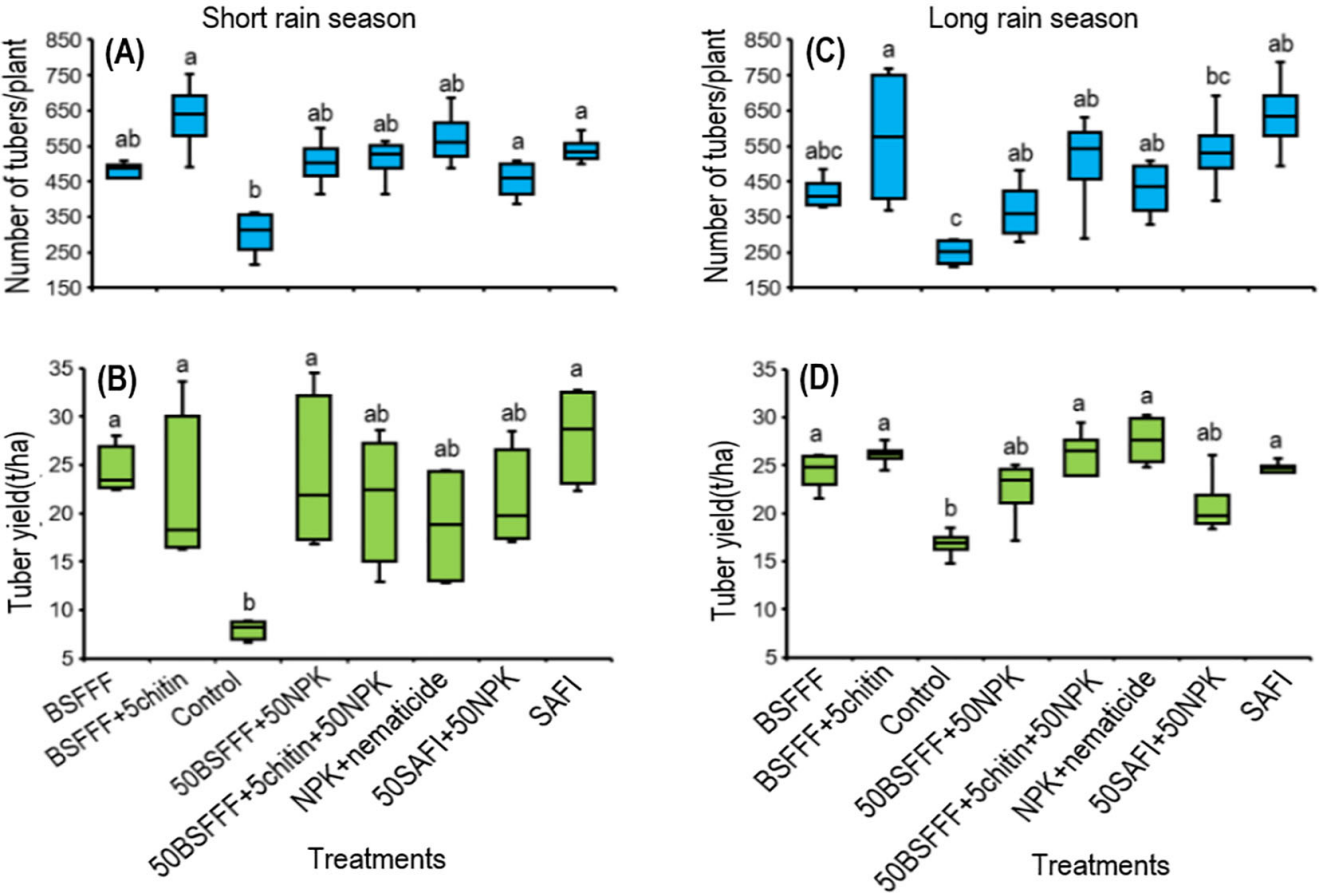
Effect of BSF frass fertilizer, commercial organic fertilizer, and NPK treatments on the number of potato tubers **(A, C)** and tuber yield **(B, D)** during the short rain season **(A, B)** and long rain season **(C , D)**. Control, no fertilizer amendment; BSFFF, black soldier fly frass fertilizer applied at a rate equivalent to 150 kg N ha^-1^; BSFFF+5chitin, black solder fly frass fertilizer applied at a rate of 150 kg N ha^-1^ and fortified with 5% chitin from BSF exuviae; 50BSFFF+50NPK, 50%black soldier fly frass fertilizer+50%NPK; 50BSFFF+5chitin +50NPK, 50%BSFFF+5%chitin+50%NPK; SAFI, Commercial organic fertilizer applied at a rate equivalent to 150 kg N ha^-1^; 50SAFI+50NPK, 50%SAFI+50%NPK; NPK+nematicide, NPK+ commercial nematicide. Per panel, means (± standard error) followed by the same letters are not significantly different at p < 0.05.

The fertilizer treatments significantly increased the number of marketable potato tubers by 42 – 61% during the short rain season (*F*_(7, 24)_ = 4.2, *P* < 0.01) and 34 – 52% during the long rain season (χ^2^ = 37.2, df =7, *P* < 0.001) compared to the control (**Figures 3A, C**). The number of marketable tubers achieved using BSFFF+5chitin was comparable to the number achieved using NPK + nematicides and SAFI during both the short rain and long rain seasons, respectively. The highest number of marketable tubers achieved using BSFFF+ 5% chitin was 9 – 28% higher than other fertilizer sources.

Soil amendments with the different fertilizer treatments significantly increased the potato tuber yield during the short rain season (*F*_(7, 24)_ = 4.1, *P* < 0.01), and long rain season (χ^2^ = 37.2, df =7, *P* < 0.001) (**Figures 3B, D**. The Fertilizer treatments increased the tuber yield by 34 – 72% and 20 – 39% compared to the control during the short and long rains, respectively. The marketable tuber yields achieved using BSFFF, 50BSFFF + 50NPK, and BSFFF+ 5chitin were comparable to the value achieved using SAFI, but higher than NPK + nematicides by 12–32% during the short rain season. During the long rain season, the yields achieved using the BSFFF formulations were comparable to the yield achieved using SAFI and NPK + nematicides.

### 3.3 Effect of chitin-fortified BSFFF and commercial fertilizers and nematicides on nematode population density and reproduction rate

The fertilizer amendments significantly (*P* < 0.001) reduced the number of cysts per 200g soil^-1^ by 23 – 50% and 32 – 58% compared to the control, during the short rain season (χ^2^ = 27.7, df =7, *P* < 0.001) and long rain season (*F*_(7, 24)_ = 24.0, *P* < 0.01), respectively (**Table 2**). Soil amendment with BSFFF+ 5chitin achieved the highest reduction, which was 5% and 35% higher than the values achieved using NPK + nematicide and SAFI respectively, during the short rain season, and 7 – 20% higher than those of SAFI treatments during the long rain season. During the long rain season, NPK + nematicide achieved the highest reduction in the number of cysts which was 24% and 33% higher than the values achieved using BSFFF+ 5chitin and SAFI, respectively.

**Table 2:** Effect of fertilizer treatments on potato cyst nematode population parameters.

Treatments	Short rains					Long rains				
	Number of cysts/200gsoil	Number of eggs and J2/cyst	Number of eggs and J2/200g-soil	Reproduction rate (*pf/pi*)	Percentage reduction	Number of cysts/200g-soil	Number of eggs/and 2/cyst	Number of eggs and J2/200g-soil	Reproduction rate (*pf/pi*)	Percentage reduction
Control	895.5 ± 46.40a	178.3 ± 15.97a	599.4 ± 106.72a	37.8 ± 6.79a	0	806.5 ± 71.8a	260.6 ± 6.90a	1050.8 ± 95.65a	30.4 ± 6.39a	0
BSFFF	533.8 ± 74.51b	60.9 ± 5.31b	160.5 ± 24.19b	18.2 ± 5.41ab	51.9	486.3 ± 48.66ab	94.2 ± 10.38bc	182.7 ± 25.42c	15.9 ± 7.33ab	47.7
BSFFF + 5% chitin	450.5 ± 44.83b	79.11 ± 7.56b	183.1 ± 34.99b	8.76 ± 3.03b	76.8	442.0 ± 63.04b	27.1 ± 1.77 c	58.7 ± 7.84c	8.07 ± 1.63ab	73.4
50BSFFF + 50NPK	466.5 ± 12.58b	97.6 ± 6.03b	228.6 ± 19.71b	14.5 ± 6.16ab	61.6	466.5 ± 50.30ab	62.4 ± 14.53bc	134.9 ± 19.14c	20.1 ± 10.24ab	33.9
50BSFFF+ 5chitin + 50NPK	525.5 ± 88.50b	106.9 ± 5.38b	278.1 ± 41.26b	34.8 ± 7.90a	7.9	514.3 ± 50.36b	120.7 ± 20.7b	324.6 ± 82.68bc	10.5 ± 3.11ab	65.4
SAFI	694.5 ± 121.76ab	115.6 ± 24.35ab	427.1 ± 130.88ab	35.1 ± 13.69a	7.1	550.5 ± 151.8ab	269.4 ± 10.52a	734.9 ± 194.15ab	23.7 ± 4.04ab	22.0
50SAFI + 50NPK	565.3 ± 112.9ab	84.12 ± 13.78b	247.3 ± 77.32b	23.7 ± 6.24ab	37.3	497.5 ± 38.42ab	135.5 ± 17.76b	346.9 ± 63.1bc	19.6 ± 6.78ab	35.5
NPK + nematicide	473.8 ± 23.68b	60.0 ± 25.68b	145.0 ± 63.0b	10.91 ± 2.87b	71.1	336.0 ± 36.24b	14.1 ± 1.25c	22.9 ± 0.8c	2.9 ± 1.0b	90.4
Significance level	***	***	***	*		**	***	***	*	

***p < 0.001, **p < 0.01, *p < 0.05, ns, non-significant; Control, no fertilizer amendment; BSFFF, black soldier fly frass fertilizer applied at a rate equivalent to 150 kg N ha^-1^; BSFFF+5chitin, black solder fly frass fertilizer applied at a rate of 150 kg N ha^-1^ and fortified with 5% chitin from BSF exuviae; 50BSFFF+50NPK, 50%black soldier fly frass fertilizer +50%NPK; 50BSFFF+ 5chitin +50NPK, 50%BSFFF+5%chitin+50%NPK; SAFI, Commercial organic fertilizer applied at a rate equivalent to 150 kg N ha^-1^; 50SAFI+50NPK, 50%SAFI+50%NPK; NPK +nematicide, NPK + commercial nematicide. Means (± standard error) followed by the same letters are not significantly different at p < 0.05

All treatments significantly reduced the number of eggs and J2 per cyst by 35 – 66% and 50 – 94% compared to the control (short rain season: *F*_(7, 24)_ = 6.3, *P* < 0.001, long rain season: χ^2^ = 431.4, df =7, *P* < 0.001), respectively (**Table 2**).

The highest reduction in the number of eggs and J2 per cyst was achieved using NPK + nematicide. However, this value was not significantly different from BSFFF based fertilizer sources. The BSFFF-based fertilizer sources reduced the number of eggs and J2s per cyst by 8 – 47% and 55 – 90% compared to SAFI during the short and long rains, respectively.

The different fertilizer treatments significantly reduced the number of cyst eggs and J2 per 200g soil^-1^ by 29 – 76% and 30 – 98% compared to the control during the short rain season (χ^2^ = 31.9, df =7, *P* < 0.001) and long rain season (χ^2^ = 123.4, df =7, *P* < 0.001), respectively (**Table 2**). NPK + nematicide achieved the highest reduction in the number of cyst eggs and J2 per 200g soil^-1^ though not significantly different from BSFFF-based fertilizer sources in both seasons. Compared to SAFI, the BSFFF-based fertilizer sources reduced the number of cyst eggs and J2 per 200g soil^-1^ by 35 – 62% and 56 – 92% during the short and long rain seasons respectively.

Soil amendment with the different fertilizer treatments caused significant differences in PCN reproduction rate. The fertilizer treatments reduced the reproduction rate by 8 – 77% and 22– 91% compared to the control during the short rain season (χ^2^ = 18.1, df =7, *P* < 0.01) and long rain season (χ^2^ = 16.3, df =7, *P* < 0.05) respectively (**Table 2**). The highest reduction in the PCN reproduction rate achieved using BSFFF+5chitin was 20% and 75% different from the values achieved using NPK + nematicide and SAFI respectively, during the short rain season. During the long rain season, soil amendment with NPK + nematicide caused the highest reduction in the PCN reproduction rate. However, this was not significantly different from the values achieved using the BSFFF-based fertilizer sources. Compared to SAFI, the BSFFF-based fertilizer sources reduced the nematode reproduction rate by 15 – 66%.

### 3.4 Multivariate analysis of potato growth, yield, and nematode population parameters

**Figure 4:**
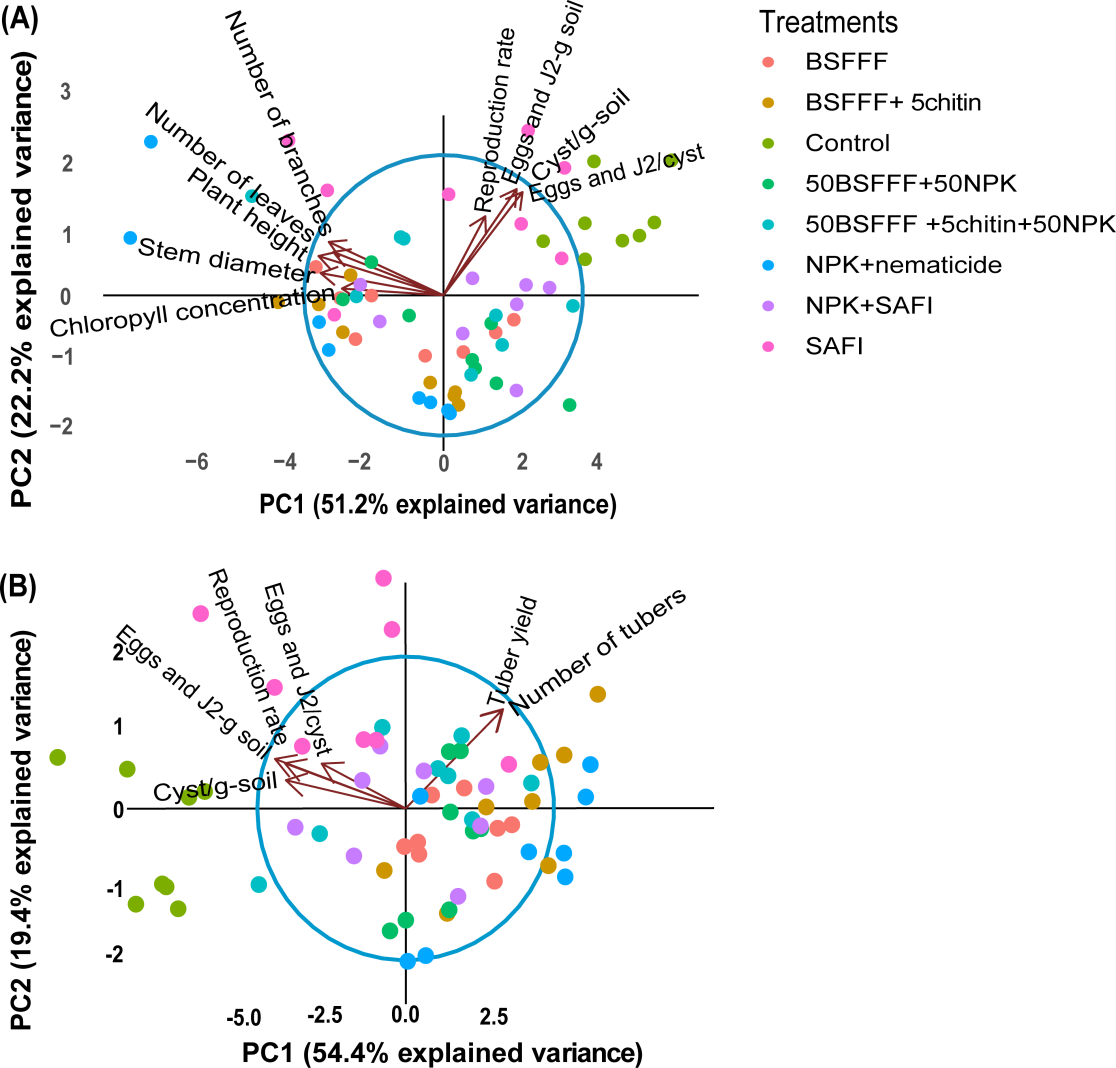
Biplots showing the relationship between nematode population parameters and potato growth parameters **(A)**, nematode population parameters, and potato yield parameters **(B)**, for the first two principal components (PC1 and PC2). Control, no fertilizer amendment; BSFFF, black soldier fly frass fertilizer applied at a rate equivalent to 150 kg N ha^-1^; BSFFF+5chitin, black solder fly frass fertilizer applied at a rate of 150 kg N ha^-1^ and fortified with 5% chitin from BSF exuviae; 50BSFFF+50NPK, 50%black soldier fly frass fertilizer+50%NPK; 50BSFFF+5chitin +50NPK, 50%BSFFF+5%chitin+50%NPK; SAFI, Commercial organic fertilizer applied at a rate equivalent to 150 kg N ha^-1^; 50SAFI+50NPK, 50%SAFI+50%NPK; NPK+nematicide, NPK+ commercial nematicide. Per panel, means (± standard error) followed by the same letters are not significantly different at p < 0.05.

The fertilizer treatments affected the potato growth, yield, and nematode population parameters as shown by the principal component analysis (PCA) (**Figure 4**). For the potato growth and nematode population parameters (**Figure 4A**), the first two components accounted for 73.4% of the total variance. PCA 1 accounted for 51.2% of the variance while PCA 2 accounted for 22.2%. The nematode population parameters were negatively correlated with the potato growth parameters. However, fertilizer treatments such as NPK + nematicide, BSFFF, and its combinations clustered towards the potato growth parameters, while the control, SAFI, and its combination with NPK clustered towards the nematode population parameters. The PCA analysis for nematode population parameters and potato yield (**Figure 4B**) showed that the first two components accounted for 73.8% of the total variance, whereby, PC 1 accounted for 54.4% and PC 2 accounted for 19.4% of the total variance. The unamended soil (control), SAFI, and its combination with NPK clustered towards the nematode population parameters while, NPK+nematicide, BSFFF, and its combinations clustered towards the potato yield parameters.

## 4 Discussion

### 4.1 Effect of chitin-fortified BSF frass fertilizer, commercial fertilizers, and nematicides on potato growth and yield

The enhanced potato growth and yield achieved using both SAFI and BSF frass fertilizers compared to the unfertilized soil indicate the high levels of soil degradation and justify the need for fertilizer application to improve soil and crop productivity (Tully et al., 2015). Therefore, the comparable values of potato growth and yield parameters achieved using commercial fertilizers (SAFI and NPK+ nematicide) and BSF frass fertilizer showed that this novel insect-based fertilizer can complement or replace commercial nematicide and fertilizers used in potato production thus, confirming the findings of our earlier study under greenhouse conditions (Anedo et al., 2024). The regenerative chitin-fortified BSFFF will go a long way in providing a holistic solution for transforming agri-food systems due to its multipurpose roles of nutrient supply and pest control.

The improved potato growth and yield observed in this study could be attributed to enhanced nutrient availability and synchrony associated with BSFFF (Beesigamukama et al., 2021; Boudabbous et al., 2023). Recent studies also demonstrated the efficacy of BSFFF or chitin-fortified BSFFF in significantly improving fertility in terms of soil pH, macronutrients, exchangeable cations, and cation exchange capacity (Beesigamukama et al., 2021; Gebremikael et al., 2022; Menino et al., 2021; Boudabbous et al, 2023; Anedo et al., 2024). On the other hand, soil amendment with BSFFF and the chitin-rich exuviae have been found to boost plant growth hormones and stimulate the activities of beneficial bacteria and fungi that are key in enhancing plant growth and immunity (Wantulla et al., 2023b; van de Zande et al., 2024). The BSFFF also provides additional benefits, especially inducing systemic resistance against pests and pathogens, attracting pollinators and parasitoids, and upregulating plant defensive genes (Barragan-Fonseca et al., 2023; Li et al., 2023; Wantulla et al., 2023a). This could have contributed to the superior performance of BSFFF-based fertilizers observed during the study. To complement the findings of Anedo et al. (2024), future studies are warranted to validate the effects of BSFFF and chitin-fortified BSFFF on soil nutrient cycling, soil microbiome, plant pathogens, soil-plant feedback mechanisms that contribute to pest control and diversity of pollinators and natural enemies.

The higher potato yield achieved validates our recent study which reported significant improvement in potato yield using chitin-fortified BSF frass fertilizers under greenhouse conditions, due to enhanced soil fertility and nematode suppression (Anedo et al., 2024). Moreover, previous studies reported increased growth and yield of different food crops grown in soils amended with insect frass fertilizer (Anyega et al., 2021; Tanga et al., 2021; Hodge and Conway, 2022; Carroll et al., 2023; van de Zande et al., 2024). The significant enhancements in potato growth and yield achieved using a combination of mineral fertilizer (NPK) and organic fertilizers (SAFI or BSFFF) validate the advantages of integrated nutrient management in crop production in terms of improved nutrient availability, soil moisture retention, and soil acidity reduction (Suh et al., 2015; Phom et al., 2018; Kafle et al., 2019; Stewart et al., 2020). Additionally, such practices have been shown to enhance the soil nitrogen cycle (Gentile et al., 2008) and improve soil biochemical properties (Zhou et al., 2022). The higher growth and yield obtained in SAFI (biochar-based fertilizer) treated soil confirm the benefits of biochar on soil fertility enhancement, carbon sequestration, and water retention, which are key for enhanced crop production (Beusch, 2021; Luis Moreno et al, 2022; Singh et al.,2022; Hu et al., 2023). Since biochar supplies less nutrients, future studies should evaluate the benefits of the combined application of biochar and insect frass fertilizer on soil health, pest control, and potato growth and yield to develop a product with the capacity to boost soil and improve crop yield.

### 4.2 Effect of chitin-fortified BSF frass fertilizer, commercial fertilizers, and nematicides on nematode management

Past studies have shown that potato cyst nematode can reduce potato growth and yield by up to 85% and 75%, respectively (Maneva and Trifonova, 2015; Berrahia and Sellami, 2022), warranting the urgent need for efficacious yet sustainable solutions. The observed reduction in the number of cysts/200 g soil^-1^, number of eggs and J2/cyst, the number of eggs and J2/200 g soil^-1^, and nematode reproduction rate in this study validate our sister studies that demonstrated the potential of BSFFF and chitin-fortified BSFFF as an environmentally friendly option for nematode management under controlled conditions (Anedo et al., 2024; Kisaakye et al., 2024). The highest reduction in the number of cysts/200 g soil^-1^ and nematode reproduction rate achieved using BSFFF+ 5% chitin, and the comparative nematode suppression rate observed between the BSFFF-based sources and NPK + nematicide highlights the potential of chitin-fortified BSFFF as a sustainable alternative or compliment to conventional nematicides used in potato production. Unlike, synthetic nematodes which are costly and pose serious health risks to both human and environmental health (Zhang et al., 2017; Sabarwal et al., 2018; Touray et al., 2021), the chitin-fortified BSFFF is bio-rational and thus contributes to safe food production and One-Health benefits.

Despite the increased nematode suppression observed in the plots treated with BSFFF, BSFFF+5chitin, and their combinations with NPK, the mechanisms involved have not been properly investigated. However, previous studies have attributed the efficacy of organic resources and chitin-rich organic soil amendments on nematode control to the high nitrogen content and low C: N ratio (Rodriguez-Kabana et al., 1987; Oka, 2010; Renčo et al., 2010). Organic amendments with low C: N ratios such as the BSFFF and chitin-fortified BSFFF used in the study have been found more effective in plant parasitic nematode suppression compared to those with high C: N ratios (Rodriguez-Kabana et al., 1987; Renčo et al., 2010). This is majorly due to the fast release of ammonia, organic acids, nitrogenous compounds, and other by-products of organic matter decomposition that significantly contribute to nematode suppression (Rodriguez-Kabana, 1986; Oka and Yermiyahu, 2002; Nico et al., 2004; Oka, 2010; Thoden et al., 2011). These compounds can either render the newly hatched juveniles immobile or alter the soil conditions in a way that prevents nematode reproduction (Oka, 2010). Soil amendment with chitin and chitin-rich organic fertilizers such as BSFFF has been reported to increase the activity and diversity of chitinolytic microbes which parasitize on nematode eggs and eggs sac, reducing their rate of reproduction and capacity to attack potato roots (Mian et al., 1982; Rodriguez-Kabana et al., 1984, Rodriguez-Kabana et al., 1987). Nyang’au et al. (2023) demonstrated the efficacy of fungal species isolated from PCN collected in different potato fields in Kenya in reducing egg viability and hatchability, contributing to bio-rational nematode control. Most of these microbes especially those belonging to the bacterial genus Pseudomonas, Streptomyces, Purpureocillium, and Pochonia, and fungi H. minnesotensis either parasitize on the nematode eggs or produce antibiotics that inhibit the action of the nematodes (Cronin et al., 1997; Devrajan et al., 2004; Mwaheb et al., 2017). For example, the antibiotic 2- 4 diacetyl phloroglucinol, produced by the bacteria P. fluorescens inhibits the activities of potato cyst nematode (G. rostochiensis) (Cronin et al., 1997). Going forward, future studies are warranted to elucidate the mechanisms of nematode suppression associated with BSFFF and chitin-fortified BSFFF to provide accurate recommendations for enhancing the efficacy of these novel bio-rationals.

The reduction in the nematode reproduction rate (91%) achieved using BSFFF+5chitin in this study is higher than the values (82 – 87%) reported by Ebrahimi et al. (2016) while using organic amendments of varying qualities. The results obtained in this study are consistent with earlier reports on the efficacy of compost, chitin, and chitin-rich organic soil amendments in nematode control (Renčo et al., 2010; Renčo and Kováčik, 2015; Ebrahimi et al., 2016; Renčo et al., 2016), and justify the use of regenerative chitin-fortified BSFFF to boost nematode control in various cropping systems. It is anticipated that long-term use of BSFFF or chitin-fortified BSFFF will increase soil suppressiveness towards PCN through residual effects, and eliminate requirements for synthetic nematicides in potato cropping systems. Farmers are, therefore, encouraged to adopt chitin-fortified BSFFF as a regenerative organic fertilizer for sustainable potato production.

Although the growth and yield of potato achieved using the commercial organic fertilizer (SAFI) in this study was comparable to BSFFF and NPK+nematicide, its nematicidal efficacy was far below the values achieved using BSFFF, BSFFF+ 5 chitin and their combinations with NPK. This is because SAFI has a high C: N ratio (53.8), low total nitrogen content (0.53%), and does not contain chitin which boosts the nematicidal properties of the BSFFF. The adoption of such fertilizers is detrimental to sustainable potato production since their continuous use would cause a surge in soil nematode populations and decrease potato yields due to high soil nematode infestation. Future studies are warranted to understand the relationship between soil nutrient availability, soil nematode population densities, potato plant performance, and tolerance to PCN. To enhance sustainable management of PCN, future studies should explore the integration of BSFFF formulations with regenerative control methods such as trap crops, endophytic fungi (Kisaakye et al., 2023), and wrap and plant technology using banana fiber paper (Ochola et al., 2022).

## 5 Conclusion

This study has demonstrated the effectiveness of chitin-fortified BSFFF as a multipurpose and regenerative organic soil amendment for improving potato growth and yield, as well as enhancing sustainable management of the devastating potato cyst nematodes. The benefits of BSFFF+5% chitin extend to sufficient nutrient supply and boost soil health, showing huge promise as a complement or substitute to conventional commercial fertilizers and nematicides currently used in potato production. The adoption of this novel strategy for soil amendment will reduce reliance on synthetic fertilizers and nematicides which are costly with harmful impacts to environmental and human health. Therefore, integrating frass fertilizer as an innovative nature-based solution geared at harnessing ecological processes to keep below-ground pests in check, maintain fertility, and prevent loss of soil nutrients and organic matter is crucial. However, multilocational field trials are warranted to validate these findings, elucidate the mechanisms of nematode suppression in soils amended with BSFFF and chitin-fortified BSFFF, determine the mid to long-term effects of BSFFF on soil health and nematode management, as well as evaluate the economic value of chitin-fortified BSFFF compared to other approaches.

## Data Availability

The original contributions presented in the study are included in the article/supplementary material. Further inquiries can be directed to the corresponding authors.
